# XIAP RING domain mediates miR-4295 expression and subsequently inhibiting p63α protein translation and promoting transformation of bladder epithelial cells

**DOI:** 10.18632/oncotarget.10645

**Published:** 2016-07-18

**Authors:** Honglei Jin, Jiheng Xu, Xirui Guo, Haishan Huang, Jingxia Li, Minggang Peng, Junlan Zhu, Zhongxian Tian, Xue-Ru Wu, Moon-Shong Tang, Chuanshu Huang

**Affiliations:** ^1^ Zhejiang Provincial Key Laboratory for Technology & Application of Model Organisms, School of Life Sciences, Wenzhou Medical University, Wenzhou, Zhejiang 325035, China; ^2^ Nelson Institute of Environmental Medicine, New York University, School of Medicine, Tuxedo, NY 10987, USA; ^3^ Department of Urology, New York University School of Medicine, New York, NY 10016, USA; ^4^ Department of Pathology, New York University School of Medicine, New York, NY 10016, USA; ^5^ VA Medical Center in Manhattan, New York, NY 10010, USA

**Keywords:** bladder cancer transformation, XIAP, RING domain, p63α

## Abstract

The X-linked inhibitor of apoptosis protein (XIAP) contains three N-terminal BIR domains that mediate anti-apoptosis and one C-terminal RING finger domain whose function(s) are not fully defined. Here we show that the RING domain of XIAP strongly inhibits the expression of p63α, a known tumor suppressor. XIAP knockdown in urothelial cells or RING deletion in knockin mice markedly upregulates p63α expression. This RING-mediated p63α downregulation is critical for the malignant transformation of normal urothelial cells following EGF treatment. We further show that the RING domain promotes Sp1-mediated transcription of miR-4295 which targets the 3′UTR of p63α mRNA and consequently inhibits p63α translation. Our results reveal a previously unknown function of the RING of XIAP in promoting miR-4295 transcription, thereby reducing p63α translation and enhancing urothelial transformation. Our data offer novel insights into the multifunctional effects of the XIAP RING domain on urothelial tumorigenesis and the potential for targeting this frequently overexpressed protein as a therapeutic alternative.

## INTRODUCTION

The X-linked inhibitor of apoptosis protein (XIAP) is a member of the IAP family and a potent inhibitor of apoptosis [1, 2]. It has been reported that XIAP is upregulated in acute and chronic leukemia [3, 4], prostate cancer [5], breast cancer [6–8], and many other cancers [9–11], thus indicating an association between XIAP overexpression and cancer development [12, 13]. Our recent studies reveal several non-apoptosis-related functions of XIAP, including upregulation of Cyclin D1, which promotes bladder cancer cell growth [14] and promotion of F-actin formation and colon cancer cell invasion [15] *via* inhibition of SUMOlation of RhoGDIα (Rho GDP-dissociation inhibitor 1) at lys-138 [16]. Other investigators have reported the association of XIAP overexpression with cancer progression, chemoresistance and poor prognosis in cancer patients [3, 9, 11, 17].

XIAP consists of four major structural domains, including three repeats of the baculovirus IAP repeat (BIR) domain at its NH2 terminus and a RING finger domain near its COOH terminus [18]. The BIR domains inhibit caspase 3, 7 and 9, thereby antagonizing apoptosis, while the RING domain exerts E3 ubiquitin ligase activity, enabling IAPs to ubiquitinize themselves, caspase-3, and caspase-7 *via* the proteasome [19–21]. More recently, we found that the BIR domains of XIAP can bind directly to E2F1 (E2F transcription factor 1) and increases its transactivation [22]. In contrast, the biological function and molecular mechanisms underlying the RING domain of XIAP are not well understood. We have demonstrated that the RING domain participates in the inhibition of RhoGDIα SUMOlation at lys-138, in turn suppressing F-actin formation and human colon cancer invasion [16]. In the current study, we show a novel function and mechanism of the action of the RING domain in the downregulation of tumor suppressor p63α protein expression where XIAP promotes the malignant transformation of urothelial cells.

The p63 protein is a member of the p53 family of transcription factors that has been shown to be important in the development of epithelial tissues. It has been shown that p63-deficient mice have several developmental defects, such as the lack of limbs, teeth and mammary glands [23]. p63 gene encodes two major isoforms by alternative promoters:TAp63 and ΔNp63, with different transcription abilities [24]. TAp63 contains a transactivation domain (TAD) and can initiate transcription of p53-regulated genes, such as p21, bax, mdm2, and other unique targets [25], whereas ΔNp63 lacks the transactivation domain (TAD) [24]. It has been reported that loss of p63 results in spontaneous tumor formation, although the mechanism underlying the tumorigenesis is not yet fully understood [26]. The p63α is the longest TA transcript variant of p63, and has been characterized as a tumor suppressor responsible for preventing cancer development [27–31]. However, most of the existing studies focused on p63α-regulated downstream effectors and much less is known about the upstream regulators of p63α. It was this lack of knowledge regarding the upstream regulators of p63α that motivated us to carry out the present studies. Our explorations led us to discover that XIAP could inhibit p63α protein translation *via* its RING domain-initiated miR-4295 expression.

## RESULTS

### XIAP inhibited p63α protein expression specifically via its RING domain in bladder epithelial cells both *in vitro* and *in vivo*

**Figure 1:**
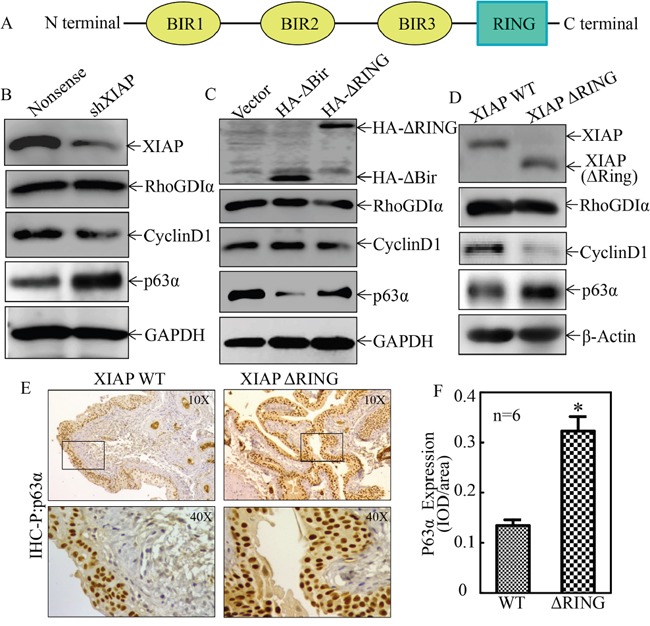
XIAP inhibited p63α expression *in vitro* and *in vivo* through its RING domain **A.** Schematic representation of XIAP protein and identified function of each domain; **B.** and **C.** The indicated cell extracts were subjected to Western blot for determination of expression of XIAP, RhoGDIα, CyclinD1 and p63α. GAPDH was used as protein loading controls; **D.** Protein extracts of mouse primary bladder epithelial cells collected from either WT-XIAP mice or XIAP-ΔRING knockin mice were subjected to Western blot for determination of expression of XIAP, RhoGDIα, CyclinD1 and p63α. β-Actin was used as protein loading controls; **E.** and **F.** IHC-P was carried out to evaluate p63α expression in mouse bladder epithelial cells obtained from WT-XIAP mice and XIAPΔRING mice. The optical density was analyzed as described in “materials and methods”. The symbol (*) indicates a significant increase in comparison to that of WT-XIAP mice (P< 0.05).

XIAP is a well-known anti-apoptosis protein with three repeat BIR domains in the N terminal and one RING domain in the C terminal as shown in Figure 1A. Our most recent studies show that RING domain of XIAP is crucial for XIAP upregulation of Cyclin D1 expression [14], while BIR domains specifically mediate E2F1 transactivation and Cyclin E expression in human colon cancer HCT116 cells [22]. We have also demonstrated that XIAP inhibits RhoGDIα SUMOylation through the RING domain using the same experimental system [16]. To evaluate the potential role of XIAP in human bladder carcinogenesis, we transfected shRNA specific targeting human XIAP into immortalized normal human bladder epithelial UROtsa cells and established the stable transfectant, UROtsa(shXIAP). As shown in Figure 1B, knockdown of XIAP resulted in profound upregulation of p63α protein expression, while it exhibited a reduction of Cyclin D1 expression, but no effect on RhoGDIα expression. These results reveal a novel function of XIAP in the suppression of p63α expression in addition to its positive regulatory effect on Cyclin D1 expression in human bladder epithelial cells. To identify the specific functional domain that are responsible for XIAP suppression of p63α, we stably transfected HA-ΔBIR and HA-ΔRING expression constructs into UROtsa cells, respectively, to establish two stable transfectants, UROtsa(HA-ΔBIR) and UROtsa(HA-ΔRING). As shown in Figure 1B, ectopic expression of HA-ΔBIR consistently inhibited p63α protein expression in UROtsa with upregulation of Cyclin D1 expression, whereas overexpression of HA-ΔRING did not show these biological effects in comparison to vector control transfectant, UROtsa(Vector) under same experimental conditions (Figure 1C). The inhibitory effect of XIAP RING domain on p63α expression was greatly supported by the results obtained from XIAP ΔRING domain knockin mice, showing that p63α expression in the bladder epithelial cells collected from XIAP ΔRING knockin mice exhibited much higher than that observed in XIAP-WT mice (Figure 1D). This finding of p63α upregulation in XIAP ΔRING knockin mice was extended to be tested *in vivo* bladder tissues from both types of mice with immunohistochemistry (IHC) staining (Figure 1E & 1F). Taken together, our results clearly demonstrate that RING domain of XIAP provides an inhibitory effect on p63α protein expression in bladder epithelial cells both *in vitro* and *in vivo*.

### p63α upregulation was crucial for XIAP RING-mediated malignant transformation of human bladder epithelial cells

**Figure 2:**
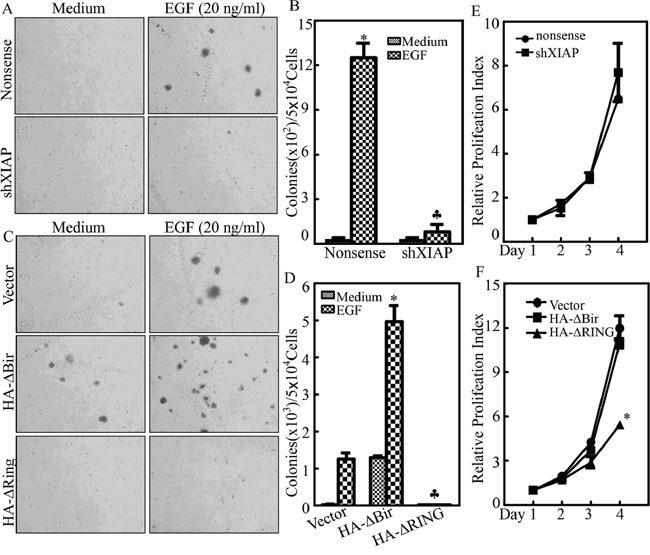
XIAP RING domain upregulated EGF induced bladder cell transformation **A.** and **B.** UROtsa(shXIAP) and UROtsa(Nonsense) cells were subjected to anchorage-independent assay in the presence or absence of EGF as indicated using the protocol described in the section of “Materials and Methods”. Representative images of colonies of UROtsa(shXIAP) and UROtsa(Nonsense) cells were captured under microscopy following 3 week incubation period; (A) the number of colonies was counted under microscopy and the results were presented as colonies per 50,000 cells from three independent experiments. The asterisk (*) indicates a significant increase in comparison to medium control and the symbol (♣) indicates a significant inhibition as compared with UROtsa(Nonsense) cells (p<0.05) (B). **C.** and **D.** UROtsa(Vector), UROtsa(HA-ΔBIR) or UROtsa(HA-ΔRING) cells were subjected to anchorage-independent assay in the presence or absence of EGF as indicated. Representative images of colonies of indicated cells were captured under microscopy following 3 week incubation period (C); the number of colonies was counted under microscopy and the results were presented as colonies per 50,000 cells from three independent experiments. The asterisk (*) indicates a significant increase as compared with UROtsa(Vector) cells, while the asterisk (♣) indicates a significant decrease as compared with UROtsa(Vector) cells (p<0.05) (D). **E.** and **F.** ATP assay to determine proliferation of cells was performed as indicated. The asterisk (*) indicates a significant inhibition as compared with UROtsa(Vector) cells.

**Figure 3:**
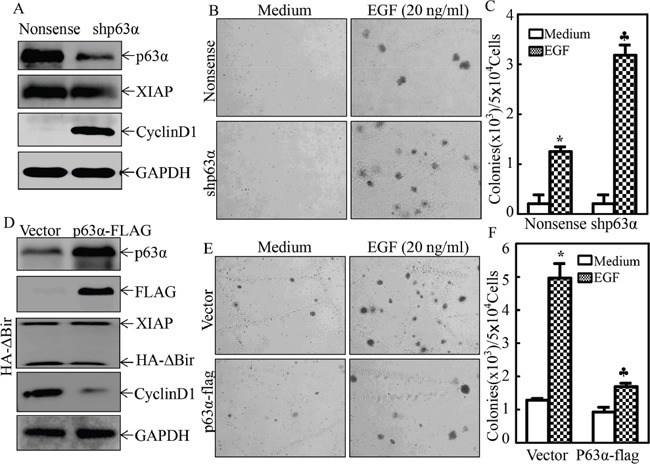
p63α inhibited EGF-induced malignant transformation of human bladder epithelial cells **A.** The cell extracts of UROtsa(Nonsense) and UROtsa(shp63α) transfectants were subjected to Western blot as indicated for identification of knockdown efficiency of shRNA targeting p63α. **B.** and **C.** UROtsa(Nonsense) and UROtsa(shp63α) transfectants were subjected to anchorage-independent growth in the presence or absence of EGF as indicated. Representative images of the colonies of indicated cells were captured under microscopy following 3 week incubation period (B); the number of colonies was counted under microscopy and the results were presented as colonies per 50,000 cells from three independent experiments. The asterisk (*) indicates a significant increase as compared with medium control, and the symbol (♣) indicates a significant increase in comparison to UROtsa(Nonsense) cells (p<0.05) (C). **D.** Western blot was used to identify the stable overexpression level of p63α-Flag in UROtsa(HA-ΔBIR) cells in comparison to UROtsa(HA-ΔBIR/Vector) control cells; **E.** and **F.** UROtsa(HA-ΔBIR/Vector) and UROtsa(HA-ΔBIR/p63α-FLAG) cells were subjected to anchorage-independent growth in the presence or absence of EGF as indicated. Representative images of the colonies of indicated cells were captured under microscopy following 3 week incubation period (E); the number of colonies was counted under microscopy and the results were presented as colonies per 50,000 cells from three independent experiments. The asterisk (*) indicates a significant increase as compared with the medium control, and the symbol (♣) indicates a significant inhibition in comparison to UROtsa(HA-ΔBIR/Vector) cells (p<0.05) (F).

Epidermal growth factor (EGF) is a well-known tumor promoter that has been widely used to induce cell transformation [32–35]. Epidermal growth factor receptor (EGFR) has been reported to be highly expressed in Bladder cancers [36]. UROtsa is a normal bladder epithelial cell line [37]. Upon exposure to bladder carcinogens, such as arsenate or cadmium, UROtsa is transformed to a malignant status, and expresses a high level of epidermal growth factor (EGF) [38]. Thus, it is thought that EGF and its receptor play an important role in bladder cancer development. To evaluate the potential role of the XIAP RING domain in malignant transformation of UROtsa cells, we exposed UROtsa cells to EGF and malignant transformative capability was evaluated by testing their anchorage-independent growth ability in soft agar. The results showed that EGF treatment did induced UROtsa cell growth in soft agar, whereas knockdown of XIAP led to almost complete abolishment of this EGF-induced malignant transformation (Figure 2A & 2B). We next tested whether the specific structural domain of XIAP was responsible for malignant transformation following EGF treatment. As shown in Figure 2C-2D, ectopic expression of HA-ΔBIR domain alone resulted in significantly increased UROtsa cell growth in soft agar, and remarkably promoted EGF-induced malignant transformation in comparison to those in UROtsa(Vector) transfectants, revealing that RING domain of XIAP exhibited strong promotion effects on both the basal level and the EGF-induced level of UROtsa cell transformation. Very interestingly, overexpression of HA-ΔRING of XIAP did not show any increase in either basal level or EGF-induced level of UROtsa cell growth in soft agar. Our results revealed that the RING domain, but not the BIR domain, mediates XIAP promoting UROtsa cell anchorage-independent growth accompanied with inhibition of p63α protein expression. It was very surprising to note that knockdown of XIAP or overexpression of HA-ΔBIR domain did not show any observable effect on UROtsa cell monolayer growth although both show regulatory effects on Cyclin D1 expression in UROtsa cells (Figure 2E & 2F). It was even more surprising to observe that introduction of the HA-ΔRING domain completely blocked EGF-induced cell transformation of UROtsa cells and profoundly inhibited monolayer growth of UROtsa cells accompanied by slight reduction of Cyclin D1 expression (Figures 1B, 2C, 2D & 2F). These results suggest that Cyclin D1 might not be a major player in RING domain-regulated cell transformation in EGF-treated UROtsa cells. We anticipate that XIAP RING domain-inhibited p63 protein expression might be associated with XIAP promotion of EGF-induced malignant transformation of UROtsa cells. To test this notion, shRNA specific targeting p63α (shp63α) was stably transfected into UROtsa cells, and the stable transfectant UROtsa(shp63α) and its vector control transfectant UROtsa(Nonsense) were established and identified as shown in Figure 3A. The knockdown of p63α led to an increase in EGF-induced malignant transformation of UROtsa cells as compared with that observed in UROtsa(Nonsense) (Figure 3B & 3C), suggesting that p63α does provide an inhibitory effect on malignant transformation induced by EGF in UROtsa cells. This notion was greatly supported by the results observed in p63α overexpressed UROtsa cells. The results showed that ectopic expression of p63α-FLAG significantly reversed HA-ΔBIR-mediated promotion of transformation in UROtsa(HA-ΔBIR/p63α-FLAG) cells following EGF treatment in comparison to that in UROtsa(HA-ΔBIR/Vector) under the same experimental conditions (Figure 3D-3F). It was noted that overexpression of p63α-FLAG did not show any effect on expression of either endogenous XIAP or exogenous HA-ΔBIR, excluding the possibility of XIAP as p63α downstream effector. Taken together, these results demonstrate that p63α downregulation plays a key role in XIAP RING domain promoting malignant transformation of UROtsa cells induced by EGF treatment.

### RING domain of XIAP inhibited p63α protein translation in human bladder UROtsa cells

**Figure 4:**
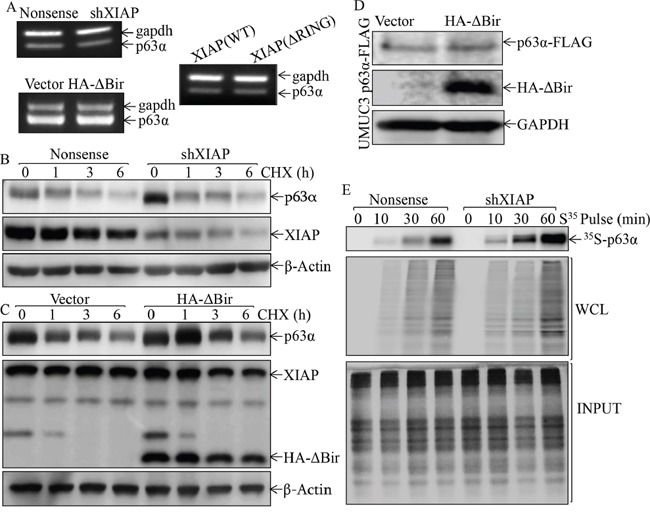
XIAP RING domain inhibited p63α protein translation in human bladder epithelial cells **A.** Total RNA was extracted from UROtsa transfectants or mouse primary bladder epithelial cells as indicated, then subjected to RT-PCR for evaluation of p63α mRNA expression. GAPDH was used as a loading control; **B.** and **C.** UROtsa(Nonsense) vs. UROtsa(shXIAP) cells (B), or UROtsa(Vector) vs. UROtsa(HA-ΔBIR) cells (C), were treated with 50 μg/ml cycloheximide (CHX) for the indicated times. The cell extracts were then subjected to Western Blot analyses of p63α protein degradation rates among the indicated cells. β-Actin was used as protein loading control. **D.** The cell extracts obtained from UMUC3(p63α-Flag/Vector) and UMUC3(p63α-Flag/HA-ΔBIR) cells were subjected to Western Blot to evaluate the effect of ectopic HA-ΔBIR expression on p63α-FLAG expression. **E.** Newly synthesized p63α protein in UROtsa(Nonsense) and UROtsa(shXIAP) cells was monitored by pulse assay using ^35^S-labeled methionine/cysteine as described in the section of “Materials and Methods”, WCL stands for whole cell lysate. Coomassie blue staining was used for protein loading control.

Our above results indicate that the XIAP RING domain inhibits p63α protein expression, which plays a critical role in RING domain promoting bladder cell malignant transformation. To elucidate the molecular mechanisms underlying the RING domain regulation of p63α expression, we first compared the mRNA levels of p63α in UROtsa(shXIAP) and UROtsa(HA-ΔBIR), with their vector control transfectants, UROtsa(Nonsense), and UROtsa(HA-ΔBIR), respectively. As shown in Figure 4A, either knockdown of XIAP or overexpression of HA-ΔBIR did not show any observable alteration of p63α mRNA expression, excluding the possibility of XIAP regulation of p63α transcription and mRNA stability in UROtsa cells. Our confidence in this finding was strengthened by the results which showed the comparable p63α mRNA expression levels between mouse bladder epithelial cells from XIAPΔRING knockin mice and WT mice (Figure 1A). Thus, we next tested to see if XIAP and its RING domain could modulate p63α protein degradation. In presence of a new protein synthesis inhibitor Cycloheximide (CHX), p63α degradation rates were evaluated between UROtsa(shXIAP) and UROtsa(Nonsense). The results showed that p63α protein degradation in UROtsa(shXIAP) cells was even greater than degradation in UROtsa(Nonsense) cells (Figure 4B). Consistent with this finding, overexpression of HA-ΔBIR profoundly delayed endogenous p63α protein degradation in UROtsa cells (Figure 4C), while overexpression of HA-ΔBIR in UMUC3 did not affect exogenous p63α-FLAG protein expression (Figure 4B). Collectively, our results exclude the possibility that XIAP and its RING domain regulate p63α at the protein degradation level. Therefore, we next tested XIAP regulation of p63α at the protein translation level. To this end, short term protein synthesis pulse-labeling assay was performed to compare p63α protein translational rates between UROtsa(Nonsense) and UROtsa(shXIAP) cells. As shown in Figure 4E, the incorporation rate of ^35^S-methionine/cysteine into newly synthesized p63α protein was gradually increased along with the incubation time periods in UROtsa(shXIAP) cells in comparison to those in UROtsa(Nonsense) cells, demonstrating that XIAP plays a crucial role in p63α protein translation in human bladder epithelial cells.

### miR-4295 expression mediated RING domain inhibition of p63α protein translation

**Figure 5:**
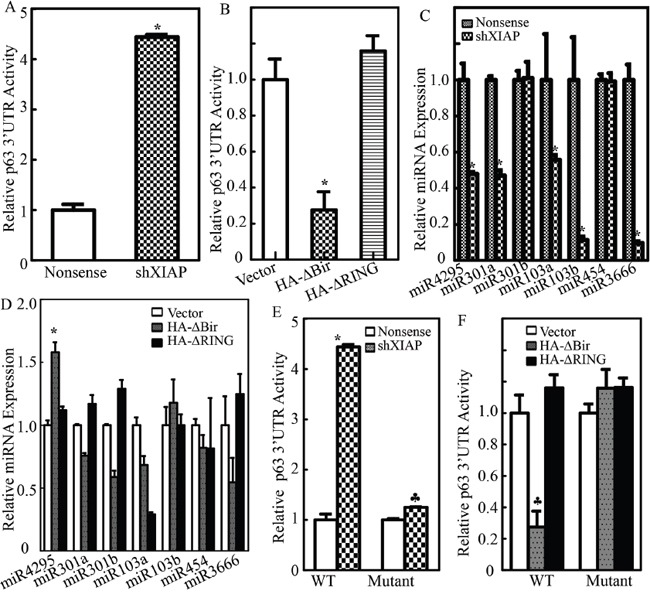
XIAP RING domain initiated miR-4295 expression and in turn inhibited p63α mRNA 3′UTR activation **A.** and **B.** Wild-type p63α 3′UTR luciferase reporters were co-transfected together with pRL-TK into UROtsa(Nonsense) vs. UROtsa(shXIAP) cells (A), or UROtsa(Vector) vs. UROtsa(HA-ΔBIR) and UROtsa(HA-ΔRING) cells (B), respectively. Twenty-four hours post transfection, the transfectants were extracted to evaluate the luciferase activity. TK was used as internal control. The results were presented as p63α 3′UTR activity relative to control vector transfectant, and each bar indicates a mean±SD from three independent experiments. The symbol (*) indicates a significant difference (P< 0.05); **C.** and **D.** The expression levels of miR-4295, miR-301a, miR-301b, miR-103a, miR-103b, miR-454 and miR-3666 were evaluated by real-time PCR as indicated. The results were normalized to U6; **E.** and **F.** UROtsa(Nonsense) vs. UROtsa(shXIAP) cells (E), or UROtsa(Vector) vs. UROtsa(HA-ΔBIR) and UROtsa(HA-ΔRING) cells (F), were transfected with either p63α 3′UTR luciferase reporter or mutant of p63α 3′UTR luciferase reporter that has an miR-4295 binding site mutation, Twenty-four hours post transfection, the transfectants were extracted to evaluate the luciferase activity. TK was used as internal control. The results were presented as p63α 3′UTR activity relative to control vector transfectant. Each bar indicates a mean±SD from three independent experiments. The symbol (*) shows a significant increase and the symbol (♣) indicates a significant inhibition (P< 0.05).

The 3-terminal untranslated region of mRNA, named 3′UTR has been reported to be involved in the regulation of its mRNA translatability [39]. To determine whether 3′UTR of p63α mRNA is involved in XIAP regulating p63α protein translation, we constructed p63α 3′UTR luciferase reporter, which was further stably transfected into two paired transfectants, UROtsa(Nonsense) *vs*. UROtsa(shXIAP) cells, or UROtsa(Vector) *vs*. UROtsa(HA-ΔBIR) and UROtsa(HA-ΔRING) cells. As shown in Figure 5A, knockdown of XIAP significantly promoted p63α 3′UTR activity, whereas ectopic expression of HA-ΔBIR, but not HA-ΔRING, dramatically inhibited p63α 3′UTR activity (Figure 5B), suggesting that the RING domain, but not the BIR domain, inhibits p63α protein translation by targeting 3′UTR of p63α mRNA. The microRNA, a class of ~22-nucletide noncoding small RNAs, has been reported to be able to bind to the 3′UTR of target mRNA and inhibits its protein translation. Therefore, we used online Targetscan 6.2 to analyze the potential miRNAs that could bind to 3′UTR of p63α mRNA. The result was shown in Table 1, and there were multiple potential miRNA binding sites, such as miR-4295, miR-301a, miR-301b, miR-103a, miR-103b, miR-454 and miR-3666, in 3′UTR of p63α mRNA. We next evaluated and compared these miRNAs expression levels in two paired transfectants, UROtsa(Nonsense) vs. UROtsa(shXIAP) cells, or UROtsa(Vector) vs. UROtsa(HA-ΔBIR) and UROtsa(HA-ΔRING) cells. The results obtained from the quantitative real-time PCR assay showed that only miR-4295 expression was consistent with its anticipated regulation of p63α mRNA 3′UTR activity in two paired transfectants, UROtsa(Nonsense) vs. UROtsa(shXIAP) cells, or UROtsa(Vector) vs. UROtsa(HA-ΔBIR) and UROtsa(HA-ΔRING) cells, showing that miR-4295 expression was profoundly inhibited in UROtsa(shXIAP) cells and was significantly upregulated in UROtsa(HA-ΔBIR) cells (Figure 5C & 5D). To provide direct evidence demonstrating the crucial role of miR-4295 binding site in p63α mRNA 3′UTR activation, we did point mutation of miR-4295 binding site in p63α 3′UTR luciferase reporter and both the WT-p63α 3′UTR luciferase reporter and its mutant were then transfected into two paired transfectants, UROtsa(Nonsense) vs. UROtsa(shXIAP) cells or UROtsa(Vector) vs. UROtsa(HA-ΔBIR) and UROtsa(HA-ΔRING) cells, respectively. The results showed that point mutation of miR-4295 binding site in p63α 3′UTR-luciferase reporter completely abolished the promotion of p63α 3′UTR activity by XIAP knockdown and also reversed the inhibition of p63α 3′UTR activity upon HA-ΔBIR overexpression (Figure 5E & 5F). These results demonstrate that miR-4295 expression mediates the RING domain inhibition of p63α protein translation by directly binding to and targeting p63α mRNA 3′UTR in UROtsa cells.

**Table 1:** The potential miRNAs binding sites in the p63α mRNA 3′UTR region

p63α: hsa- miR-4295:	5′ ..(406) UGAAAGAAAAUUGAGUUGCACUU (429)… | | | | | | | 3′ UUCCUUUUGUAACGUGAC
p63α: hsa-miR-301a:	5′ ..(406) UGAAAGAAAAUUGAGUUGCACUU (429)… | | | | | | | 3′ CGAAACUUAUGAUAACGUGAC
p63α: hsa- miR-301b:	5′ …(406) UGAAAGAAAAUUGAGUUGCACUU (429)… | | | | | | | 3′ CGAAACUGUUAUAGUAACGUGAC
p63α: hsa- miR-103a:	5′ …(406) UGAAAGAAAAUUGAGUUGCACUU (429)… | | | | | | | 3′ UACGGGAAAAUUGUAACGUGAC
p63α: hsa- miR-103b:	5′ …(406) UGAAAGAAAAUUGAGUUGCACUU (429)… | | | | | | | 3′ UACGGGAAAAUUGUAACGUGAC
p63α: hsa- miR-454:	5′ …(406) UGAAAGAAAAUUGAGUUGCACUU (429)… | | | | | | | 3′ UGGGAUAUCGUUAUAACGUGAU
p63α: hsa- miR-3666:	5′ …(406) UGAAAGAAAAUUGAGUUGCACUU (429)… | | | | | | | 3′ AGCCGUGAUGUGAACGUGAC

**Figure 6:**
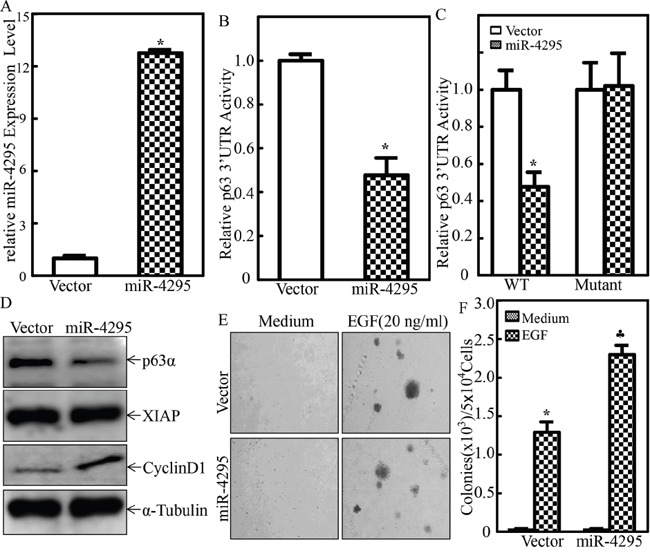
miR-4295 was able to directly bind to p63α 3′UTR resulting in blockage of p63α protein translation and subsequently promoting EGF-induced malignant transformation of human bladder epithelial cells **A.** Real-time PCR was used to identify the expression level of miR-4295 in UROtsa cells; **B.** p63α 3′UTR luciferase reporters were co-transfected together with pRL-TK into UROtsa(Vector) and UROtsa(miR-4295) cells, respectively. Twenty-four hours post transfection, the transfectants were extracted for measurement of the luciferase activity, while TK was used as internal control. The results were presented as p63α 3′UTR activity relative to vector control transfectant, and each bar indicates mean±SD from three independent experiments. The symbol (*) shows a significant difference (P< 0.05); **C.** UROtsa(Vector) and UROtsa(miR-4295) were transfected with either p63α 3′UTR luciferase reporter or mutant of p63α 3′UTR luciferase reporter that has miR-4295 binding site mutation, Twenty-four hours post transfection, the transfectants were extracted to evaluate the luciferase activity, with TK used as an internal control. The results were presented as p63α 3′UTR activity relative to control vector transfectant. Each bar indicates mean±SD from three independent experiments. The symbol (*) shows a significant difference (P<0.05); **D.** The cell extracts obtained from UROtsa(Vector) and UROtsa(miR-4295) cells were subjected to Western blot to determine expression of p63α and XIAP, while α-Tubulin was used as a protein loading control; **E.** and **F.** UROtsa(Vector) and UROtsa(miR-4295) transfectants were subjected to anchorage-independent growth in the presence or absence of EGF as indicated. Representative images of the colonies of indicated cells were captured under microscopy following 3 week incubation period (E); the number of colonies was counted under microscopy and the results were presented as colonies per 50,000 cells from three independent experiments. The asterisk (*) indicates a significant increase as compared with the medium control, and the symbol (♣) indicates a significant inhibition in comparison to UROtsa(Vector) cells (p<0.05) (F).

To evaluate the biological effect of miR-4295 on p63α mRNA 3′UTR activation, protein expression and malignant cell transformation of human bladder epithelial cells, we constructed a miR-4295 overexpression plasmid, and the construct was then stably transfected into UROtsa cells together with p63α mRNA 3′UTR luciferase reporter. The overexpression of miR-4295 in UROtsa cells was identified by quantitative real-time PCR, showing over 12-fold expression of miR-4295 in comparison to vector control transfectant (Figure 6A). Ectopic expression of miR-4295 resulted in a significant inhibition of p63α 3′UTR activity (Figure 6B); this inhibition of p63α 3′UTR activity by miR-4295 was not observed in the transfectant of p63α 3′UTR luciferase reporter with miR-4295 binding site mutant (Figure 6C), revealing that miR-4295 inhibition of p63α 3′UTR activity depends on miR-4295 binding site of p63α 3′UTR luciferase reporter. Consistently, overexpression of miR-4295 impaired p63α protein expression (Figure 6D) accompanied with the promoting of EGF-induced malignant transformation of human epithelial UROtsa cells (Figure 6E & 6F). Collectively, our results demonstrate that RING domain of XIAP is able to initiate miR-4295 expression, which subsequently binds to p63α mRNA 3′UTR, and inhibits p63α protein translation, in turn promoting EGF-induced malignant transformation of UROtsa cells.

### RING domain of XIAP promoted miR-4295 transcription by upregulating Sp1 protein expression and transcriptional activation

**Figure 7:**
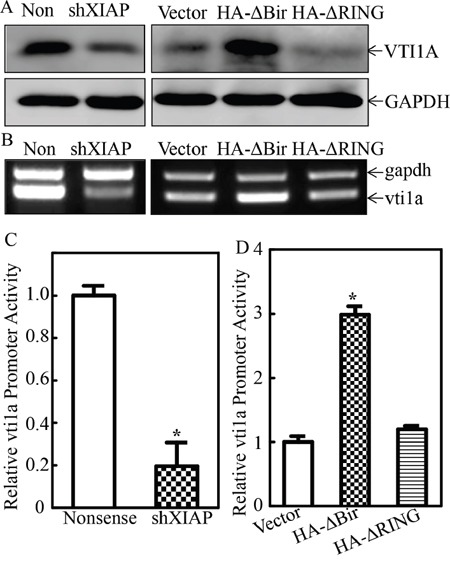
XIAP RING domain promoted miR-4295 host gene VTI1A transcription in human bladder epithelial cells **A.** and **B.** The cell extracts were subjected to Western Blot to determine expression levels of VTI1A protein (A) and mRNA (B) in UROtsa(Nonsense) vs. UROtsa(shXIAP) cells (A); or UROtsa(Vector) vs. UROtsa(HA-ΔBIR) and UROtsa(HA-ΔRING) cells as indicated. **C.** and **D.** VTI1A promoter-driven luciferase reporter was transfected together with TK reporter into UROtsa(Nonsense) vs. UROtsa(shXIAP) cells (C), or UROtsa(Vector) vs. UROtsa(HA-ΔBIR) and UROtsa(HA-ΔRING) cells (D). Twenty-four hours post transfection, the transfectants were extracted to evaluate the luciferase activity, with TK used as internal control. The results were presented as luciferase activity relative to vector control transfectant. Each bar indicates mean±SD from three independent experiments. The symbol (*) shows a significant difference (P<0.05).

To explore the mechanisms underlying RING domain regulation of miR-4295 expression, the host gene of miR-4295 was identified by searching NCBI. The results showed that the precursor of miR-4295 was encoded by the intron region of vesicle transport through interaction with t-SNAREs homolog 1A (VTI1A) gene. Thus, we first evaluated the effect of XIAP and its RING domain on VTI1A expression. As shown in Figure 7A, knockdown of XIAP impaired VTI1A expression, while overexpression of HA-ΔBIR, but not HA-ΔRING, exhibited an increase VTI1A expression at both protein and mRNA levels (Figure 7A & 7B). To test the possibility that XIAP and its RING regulate VTI1A transcription, the VTI1A promoter-drive luciferase reporter was constructed and transfected into two paired transfectants, UROtsa(Nonsense) vs. UROtsa(shXIAP) cells, or UROtsa(Vector) vs. UROtsa(HA-ΔBIR) and UROtsa(HA-ΔRING) cells, respectively. The results indicated that VTI1A promoter-driven luciferase reporter transcription activity was significantly downregulated in UROtsa(shXIAP) cells and upregulated in UROtsa(HA-ΔBIR) cells, demonstrating that XIAP and its RING domain is essential for VTI1A transcription level (Figure 7C and 7D).

**Figure 8:**
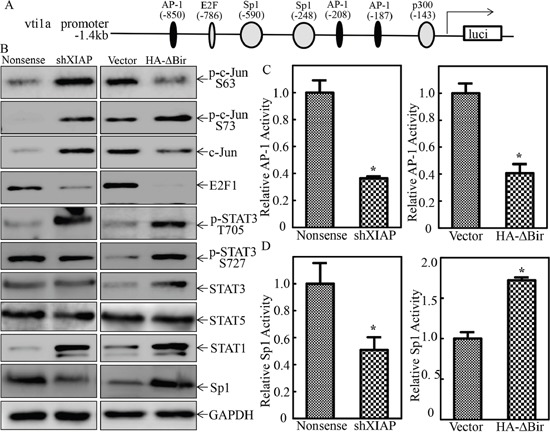
XIAP RING domain was crucial for Sp1 protein expression and transactivation in human bladder epithelial cells **A.** The potential transcription factors binding sites of VTI1A promoter. **B.** The cell extracts obtained from UROtsa(Nonsense), UROtsa(shXIAP), UROtsa(Vector), and UROtsa(HA-ΔBIR) cells were subjected to Western Blot for determination of expression of p-c-Jun S63, p-c-Jun S73, c-Jun, E2F1, p-STAT3 705, p-STAT3 727, STAT3, STAT5, STAT1 and Sp1 as indicated. **C.** and **D.** UROtsa(Nonsense), UROtsa(shXIAP), UROtsa(Vector), and UROtsa(HA-ΔBIR) cells were co-transfected with AP-1 luciferase reporter or Sp1 luciferase reporter, together with pRL-TK. Twenty-four hours post transfection, the transfectants were extracted to evaluate the luciferase activity, with normalization to TK. The results were presented as luciferase activity relative to vector control transfectant. Each bar indicates mean±SD from three independent experiments. The symbol (*) shows a significant difference (P<0.05).

**Figure 9:**
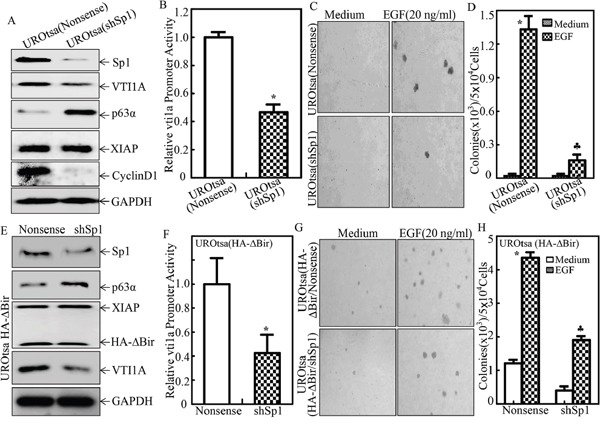
Sp1 was crucial for VTI1A transcription, p63α expression and EGF-induced malignant transformation of human bladder epithelial cells **A.** The cell extracts obtained from UROtsa(Nonsense), and UROtsa(shSp1) were subjected to Western Blot for determination of expression of protein expression as indicated; **B.** UROtsa(Nonsense) and UROtsa(shSp1) were co-transfected with VTI1A promoter-driven luciferase reporter and pRL-TK. Twenty-four hours post transfection, the transfectants were extracted to evaluate the luciferase activity, with normalization to TK. The results were presented as luciferase activity relative to nonsense control transfectant. Each bar indicates mean±SD from three independent experiments. The symbol (*) shows a significant decrease as compared to UROtsa(Nonsense) cells (P<0.05); **C.** and **D.** UROtsa(Nonsense) and UROtsa(shSp1) were subjected to anchorage-independent growth in the presence or absence of EGF as indicated. Representative images of the colonies of indicated cells were captured under microscopy following 3 week incubation period (C); the number of colonies was counted under microscopy and the results were presented as colonies per 50,000 cells from three independent experiments. The asterisk (*) indicates a significant decrease as compared with the medium control, and the symbol (♣) indicates a significant inhibition in comparison to UROtsa(Nonsense) cells (p<0.05) (D). **E.** The cell extracts obtained from UROtsa(HA-ΔBIR/Nonsense), and UROtsa(HA-ΔBIR/shSp1) were subjected to Western Blot to determine expression of protein expression as indicated; **F.** UROtsa(HA-ΔBIR/Nonsense) and UROtsa(HA-ΔBIR/shSp1) were co-transfected with vti1a promoter-driven luciferase reporter and pRL-TK. Twenty-four hours post transfection, the transfectants were extracted to evaluate the luciferase activity, with normalization to TK. The results were presented as luciferase activity relative to nonsense control transfectant. Each bar indicates mean±SD from three independent experiments. The symbol (*) shows a significant decrease as compared to UROtsa(HA-ΔBir/Nonsense) cells (P<0.05); **G.** and **H.** UROtsa(HA-ΔBIR/Nonsense) and UROtsa(HA-ΔBIR/shSp1) were subjected to anchorage-independent growth in presence or absence of EGF as indicated. Representative images of colonies of indicated cells were captured under microscopy following 3 week incubation period (G); the number of colonies was counted under microscopy and the results were presented as colonies per 50,000 cells from three independent experiments. The asterisk (*) indicates a significant decrease as compared with the medium control, and the symbol (♣) indicates a significant inhibition in comparison to UROtsa(HA-ΔBIR/Nonsense) cells (p<0.05).

miRNA transcription is modulated by RNA Pol II-associated transcription factors and epigenetic regulators [40]. To explore the transcription factor that might be involved in transcriptional regulation of VTI1A and miR-4295, the VTI1A promoter-driven luciferase reporter was bioinformatically analyzed. As shown in Figure 8A, the promoter contains the binding sites of multiple transcription factors, including c-Jun, E2F1, Sp1, STAT3 (Signal transducer and activator of transcription 3), STAT1 (Signal transducer and activator of transcription 1) and STAT5 (Signal transducer and activator of transcription 5). To identify the specific transcription factors implicated in XIAP and its RING domain promotion of VTI1A transcription, we evaluated the expression of these transcription factors in two paired transfectants, UROtsa(Nonsense) vs. UROtsa(shXIAP) cells, and UROtsa(Vector) vs. UROtsa(HA-ΔBIR) cells. Comparison of the results obtained from these two paired cell transfectants, revealed that only alteration of Sp1 and c-Jun/c-Jun Ser63 was consistent with XIAP and its RING domain regulation of VTI1A and miR-4295 (Figure 8B). The results obtained from investigating AP-1-dependent transcription activity in two paired transfectants, UROtsa(Nonsense) vs. UROtsa(shXIAP) cells, and UROtsa(Vector) vs. UROtsa(HA-ΔBIR) cells, showed that AP-1 transactivation was inhibited in both UROtsa(shXIAP) and UROtsa(HA-ΔBIR) cells, which was not consistent with their effects on the transcriptional regulation of VTI1A and miR-4295, in turn further excluding involvement of AP-1/c-Jun in transcriptional regulation of VTI1A and miR-4295 (Figure 8C). We noted with great interest that Sp1-dependent transcription activity was downregulated in UROtsa(shXIAP), while it was upregulated in UROtsa(HA-ΔBIR) cells, consistently supporting the Sp1 transcriptional regulation of VTI1A and miR-4295 (Figure 8D). To test this notion, shRNA specific targeting Sp1 was stably transfected into UROtsa cells. The stable transfectants were established and identified as shown in Figure 9A. The knockdown of Sp1 dramatically inhibited VTI1A protein expression accompanied by p63α induction (Figure 9A). Consistent with the protein expression, VTI1A promoter-driven luciferase activity was downregulated in Sp1 knockdown transfectant, UROtsa(shSp1) (Figure 9B) and EGF-induced malignant transformation of UROtsa cells was almost completely abolished (Figure 9C & 9D). To further determine whether RING-induced cell transformation was specifically mediated by Sp1, The stable transfectant UROtsa(HA-ΔBIR/shSp1) was established by co-transfecting shRNA specifically targeting Sp1 together with vti1a promoter-driven luciferase reporter into UROtsa(HA-ΔBIR) cells as shown in Figure 9E. As anticipated, the deficiency of Sp1 in UROtsa(HA-ΔBIR) cells resulted in attenuation of vti1a promoter transcriptional activity caused by HA-ΔBIR overexpression (Figure 9F), which consequently profoundly inhibited EGF-induced cell transformation (Figure 9G & 9H). Collectively, our results demonstrate that XIAP and its RING domain can initiate Sp1-dependent transcription of VTI1A/miR-4295 expression, which subsequently inhibits p63α translation by targeting its mRNA 3′-UTR, in turn promoting malignant transformation of human bladder epithelial cells.

## DISCUSSION

Biochemical and structural studies indicate that XIAP is a protein with three BIR domains and a RING-finger domain [18]. XIAP has been reported to be upregulated in many cancers, including acute and chronic leukemia [3, 4], prostate cancer [5], breast cancer [6–8], and many other cancers [9–11]. Our most recent studies have also shown that XIAP expression is overexpressed in human bladder cancers, revealing a crucial association between XIAP and human bladder cancer development (Data not shown). However, the molecular mechanisms and specific XIAP functional domain(s) responsible for XIAP regulation of human bladder carcinogenesis has not been explored. In this study, we show that XIAP and its RING domain specifically provide an inhibitory effect on p63α protein expression both *in vitro* human bladder epithelial UROtsa cells and *in vivo* normal mouse bladder epithelial cells obtained from XIAP ΔRING knockin mice. Further studies reveal that RING of XIAP is the structural functional domain responsible for this inhibition by initiating Sp1-mediated transcription of miR-4295, which is able to bind to 3′-UTR of p63α mRNA and blocks p63α protein translation. Moreover, our studies demonstrate that the miR-4295 induction/p63α downregulation by RING domain of XIAP plays a disposable role in malignant transformation of human bladder epithelial cells following EGF treatment. These findings help us to understand the association and mechanisms of XIAP overexpression with bladder cancer development, they also enable us to explore the utilization of RING domain as a potential therapeutic target for treatment of bladder cancer patients.

Although endogenous TAp63 proteins are barely detectable in normal somatic cells, their alterations are still reported in various types of cancer tissues [41]. In comparison with well-characterized p63α downstream targets and mechanisms involved in its tumor suppressive effects, the upstream regulators for control of the p63 expression have been much less thoroughly explored, although p63 levels are known to be regulated by protein phosphorylation/ubiquitin-dependent proteasome degradation and miRNAs [42]. p63 protein stability is regulated by the ubiquitin–proteasome system [43]. For example, p53-induced E3 ubiquitin ligase Pirh2 can directly bind to p63 and cause its poly ubiquitination and degradation in keratinocytes [44]. Another E3 ubiquitin ligase Ring1B has also been shown to target p63 [45]. To the best of our knowledge, there are only a few reports showing miRNAs involvement in the regulation of p63 protein expression [46]. miR-203 can repress p63 expression in supra-basal epithelial cells, contributing to definition of the border between progenitors and differentiated epithelial cells [47]. miR-302 has also been reported to suppress p63 expression in germ cells [48], while iASPP protein inhibits the expression of miR-574-3p and miR-720, which in turn inhibits p63 expression [49, 50]. In our current studies, we discovered that XIAP and its RING domain were able to inhibit p63α protein expression in bladder epithelial cells *in vitro* human cell culture model and *in vivo* mouse bladder tissues. By excluding the possibility of XIAP/RING regulation of p63α transcription and mRNA stability, as well as protein degradation, we were able to identify the regulation of p63α protein expression by the XIAP/RING domain and identify that it occurred at translation level by probing new p63α protein synthesis using [^35^S] methionine pulse assays. We further found that 3′UTR of p63α mRNA was targeted by miR-4295, which was upregulated by XIAP/RING domain. These new discoveries provide us with significant insight into understanding the upstream regulators for control of the p63 expression in the regulation of human bladder epithelial carcinogenesis.

Micro-RNAs (miRNAs) are non-coding RNAs involved in the post-transcriptional regulation of gene expression in cellular organisms by affecting mRNAs stability and translation [51]. The miRNA expression is tightly regulated at multiple levels, including at the levels of miRNA transcription, maturing, modification by RNA editing, RNA methylation, uridylation and adenylation, argonaute loading, and RNA decay [40]. Although miR-4295 could be found in the NCBI Gene database, nothing is known about its transcriptional promoter, upstream regulators/downstream effector(s). In humans, the majority of miRNAs are encoded by either introns or exons of transcripts [52]. Our studies here indicated that VTI1A is the host gene of miR-4295 and that precursor mRNA of miR-4295 is located in the intron region of VTI1A. We also found that the RING domain, but not the BIR domain, of XIAP, was able to regulate transcription of VTI1A/miR-4295, while transcription factor Sp1 expression/transactivation was regulated by the RING domain of XIAP, and was required for XIAP/RING-mediated miR-4295 expression. The matured miRNA is incorporated into a RNA-induced silencing complex (RISC), which recognizes target mRNAs through imperfect base pairing with the miRNA and consequently results in either translational inhibition or alteration of the targeted mRNA stability [51]. In this study, we found that miR-4295 as an oncogenic miRNA could target tumor suppressor gene p63α protein expression and promotes malignant transformation of human bladder epithelial UROtsa cells. These studies demonstrate novel information related to the host gene, transcriptional promoter, and upstream regulators/downstream effector(s) of miR-4295, which provide a link between XIAP overexpression and bladder epithelial carcinogenesis.

In summary, the current studies discover that XIAP/RING domain initiates Sp1-mediated miR-4295 transcription, which directly targets the 3′UTR of p63α mRNA and inhibits p63α protein translation, and further in turn promoting transformation of human bladder epithelial cells. The complete elucidation of the XIAP RING domain in the initiating of Sp1 expression/transactivation and new effector(s) of miR-4295 is next goals of undergoing projects in our group and we will test this regulation pathway in other kind of tumors in the future. Our novel findings of XIAP RING domain in regulation of Sp1/miR4259/p63α cascade as well as identifying the crucial role of this cascade in malignant transformation of human bladder epithelial cell, may help us to utilize miR-4295 as biomarkers for the diagnosis and/or targets for the prevention and therapy of human bladder cancers.

## MATERIALS AND METHODS

### Cell lines, plasmids, and antibodies

UROtsa cell line was normal human bladder epithelial cells kindly provided by Dr. Scott H. Garrent (University of North Dakota, Grand Forks, ND), and was cultured in RPIM1640 with 10% FBS [37]; UMUC3 was a high grade human bladder cancer cell line described in our previous publications [53, 54]. It was cultured in DMEM with 10% FBS (ATLANTA, Flowery Branch, GA). The shRNA that specifically targets human XIAP, Sp1 and p63α was purchased from Open Biosystems (GE, Pittsburgh, PA). The p63α-Flag expression plasmid was bought from Addgene (Cambridge, MA). HA-ΔBIR and HA-ΔRING expression plasmids were described in our previously studies [15, 16, 22]. AP-1- and Sp1-dependent luciferase reporters were described in our previous papers [55–57]. The expression plasmid containing hsa-miR-4295 was cloned into pmR-ZsGreen1 Vector using BglII and KpnI restriction endonuclease using primers: forward 5′-GGA AGA TCT AGG ATC ACA GTT AAC TCA GAA T-3′, reverse 5′-CGG GGT ACC GCA CAA TCC AAA ACA AGA A-3′. The plasmid containing luciferase reporter under control of human VTI1A gene promoter was constructed into PGL3-BASIC vector using the primers: forward 5′-CCG CTC GAG CTA GGA GAA TCC AGT GTC-3′, reverse 5′-CCC AAG CTT TGC TTC CGG AGG GAG GTT T-3′. The plasmid containing luciferase reporter under control of human WT p63α mRNA 3′UTR were constructed into pMIR-report vector using the primers: forward 5′-TAA CTC GAG GCC TCA CCA TGT GAG CTC TTC CTA-3′, reverse 5′-GGC TCG ACT AGT ACC AAC ACT GAG AAA AGA ATA-3′. The primers for cloning mutant of p63α mRNA 3′UTR luciferase reporter are: forward 5′-AAA TGA AAG AAA ATT GAG ACT TAT TGA CCA TTT TTT AA-3′, reverse 5′-TTA AAA AAT GGT CAA TAA GTC TCA ATT TTC TTT CAT TT-3′. The specific antibodies against p63α were bought from Genetex (Invine, CA). Anti-XIAP antibody was purchased from BD (Franklin Lakes, NJ). Antibodies specific against HA, p-STAT3 Ser727, p-STAT3 Tyr705, STAT3, STAT5, and GAPDH, were purchased from Cell Signaling (Beverly, MA). Antibodies specific for VTI1A, Sp1, E2F1, β-Actin and α-Tubulin, were from Santa Cruz (Dallas, TX).

### Cell transfection

Cell transfections were performed with PolyJet™ DNA *in Vitro* Transfection Reagent (SignaGen Laboratories, Rockville, MD) according to the manufacturer's instructions. For stable transfection, UROtsa cells were subjected to selection with hygromycin B (30 μg/mL), G418 (300 μg/mL) or puromycin (0.5 μg/mL) depending on the different antibiotic resistance plasmids transfected. The cells surviving from the antibiotics selection were pooled as mass stable transfectants. UMUC3 overexpressing p63α-FLAG were selected by hygromycin (300 μg/mL).

### Western blot analysis

Whole cell extracts were prepared with the cell lysis buffer (10 mM Tris-HCl, pH 7.4, 1% SDS, and 1 mM Na_3_VO_4_) as described in our previous studies [57, 58]. Protein extracts were subjected to Western Blot with the indicated primary antibodies, and probed with the AP-conjugated secondary antibody together with the enhanced chemifluorescence system as described in a previous reports [57, 58]. The images were acquired by scanning with the PhosphorImager Typhoon FLA 7000 (GE, Pittsburgh, PA).

### Immunohistochemistry paraffin (IHC-P) of mouse bladder specimens

Mouse bladder tissues obtained from the sacrificed mice were formalin-fixed and paraffin-embedded. The antibodies specific against p63α (Genetex, CA) were used for immunohistochemical (IHC) staining as described in our previous publications [57, 59]. The resulting immunostaining images were captured using the AxioVision Rel.4.6 with computerized image analysis system (Carl Zeiss, Oberkochen, Germany). Protein expression levels were analyzed by calculating the integrated optical density per stained area (IOD/area) using Image-Pro Plus version 6.0 (Media Cybernetics, MD).

### Luciferase reporter assay

p63α mRNA 3′UTR luciferase reporter, VTI1A promoter-driven luciferase reporter, Sp1-dependent transcriptional activation luciferase reporter or AP-1-dependent transcriptional activation luciferase reporter and pRL-TK, were transiently transfected into UROtsa(shXIAP), UROtsa (HA-ΔBIR), UROtsa (HA-ΔRING) and their related control vector transfectants. Twenty-four hours after the transfection, luciferase activity was determined using the luciferase assay system kit purchased from Promega (Madison, WI,) as described in our previous studies [60, 61]. The results were normalized by internal TK signal. The results are expressed as mean±SD error from triplicate experiments.

### RT-PCR

Total RNA was extracted using the TRIzol reagent (Invitrogen, Grand Island, NY,) as described in the manufacturer's instructions and our previous studies [62, 63]. 5 μg total RNA was used for first-strand cDNA synthesis with oligdT primer by SuperScript™ III First-Strand Synthesis system (Invitrogen, Grand Island, NY) and described in our previous studies [64]. Specific primers (Invitogen, Grand Island, NY,) used for PCR amplification were: *human p63α*, forward 5′-aga cat gaa tgg act cag cc-3′, reverse 5′-ctc aat ctg ata gat ggt ggt-3′; *human vti1a*, forward 5′-aga act gct tga aca gat gga ttt g-3′, reverse 5′-agg agc tca ttc cgt act tcg tca-3′; and *human gapdh*, forward 5′-gat gat ctt gag gct gtt gtc-3′, reverse 5′-cag ggc tgc ttt taa ctc tg-3′.

### Quantitative RT-PCR

The cells used for total RNA extraction using miRNeasy Mini Kit (QIAGEN, Valencia, CA). 1 μg total RNA was used for reverse transcription. Analysis of miR-4295, miR-301a, miR-301b, miR-130a, miR-130b, miR-454, and miR-3666 expression were carried out using the miScript PCR system (QIAGEN, Valencia, CA) by 7900HT Fast Real-time PCR system (Applied Biosystems, Foster City, CA). The primer for miR-4295 assay was purchased from QIAGEN (Valencia, CA), and U6 (Invitrogen, Grand Island, NY) was used as internal control. The initial activation was performed at 95°C for 15 min, followed by 40 cycles (denaturation at 95°C for 15s, annealing at 55°C for 30s and extension at 72°C for 30s). The data were analyzed as described in the previous publication [58].

### [^35^S] methionine pulse assays

UROtsa(Nonsense) and UROtsa(shXIAP) were incubated with methionine–cysteine-free DMEM (Gibco-BRL, Grand Island, NY) containing 2% dialyzed fetal calf serum (Gibco-BRL, Grand Island, NY) in presence of MG132 (50 μM) for 1 hours. The cells were then incubated with 2% fetal bovine serum methionine–cysteine-free DMEM containing ^35^S-labeled methionine/cysteine (250 mCi per dish, Trans ^35^S-label; Perkin Elmer, Boston, MA) for the indicated time periods. The cells were extracted with lysis buffer (Cell Signaling, Beverly, MA) containing complete protein inhibitor mixture (Roche, Branchburg, NJ) on ice for 10 mins. Total lysate of 500 mg was incubated with anti-p63α antibody-conjugated agarose beads (R&D Systems, Minneapolis, MN) at 4°C overnight. The immunoprecipitated samples were washed with the cell lysis buffer five times, heated at 100°C for 5 min and then subjected to sodium dodecyl sulfate–polyacrylamide gel electrophoresis analysis. The ^35^S-labeled p63α protein was detected with the PhosphorImager Typhoon FLA 7000 (GE, Pittsburgh, PA).

### Anchorage-independent growth assay

The anchorage-independent growth ability of human bladder epithelial UROtsa and its transfectants following EGF treatment was determined in soft agar as described in our previous studies [56, 65]. Briefly, 3 ml of 0.5% agar in basal modified Eagle's medium supplemented with 10% FBS with or without EGF, respectively, was layered onto each well of 6-well tissue culture plates. One ml of UROtsa cells or their transfectants (5×10^4^) was mixed with 0.5% agar BMEM (1:3) supplemented with 10% FBS with or without EGF, then layered on top of the 0.5% agar layer in each well of 6-well tissue culture plates. Plates were incubated at 37°C in 5% CO_2_ for 2–3 weeks. The colonies with more than 32 cells were scored and are presented as colonies/10^4^ cells.

### Statistical analysis

The student's t-test was used to determine the significance between treated and untreated group. The results are expressed as mean±SD from at least three independent experiments. P<0.05 was considered to be a significant difference between compared groups.

## Abbreviations

CHX: Cycoheximide
EGF: Epidermal growth factor
EGFR: Epidermal growth factor Receptor
E2F1: E2F Transcription Factor 1
miRNA: microRNA
RhoGDIα: Rho GDP-dissociation inhibitor 1
Sp1: Specificity Protein 1
STAT1: Signal transducer and activator of transcription 1
STAT3: Signal transducer and activator of transcription 3
STAT5: Signal transducer and activator of transcription 5
UTR: Untranslated region
VTI1A: Vesicle Transport Through Interaction With T-SNAREs homolog 1A
XIAP: X-linked inhibitor of apoptosis protein

## References

[1] Eckelman BP, Salvesen GS, Scott FL (2006). Human inhibitor of apoptosis proteins: why XIAP is the black sheep of the family. EMBO reports.

[2] Caron de Fromentel C, Aberdam E, Aberdam D (2012). The two faces of p63 Janus of the p53 gene family. Medecine sciences.

[3] Akyurek N, Ren Y, Rassidakis GZ, Schlette EJ, Medeiros LJ (2006). Expression of inhibitor of apoptosis proteins in B-cell non-Hodgkin and Hodgkin lymphomas. Cancer.

[4] Byrd JC, Kitada S, Flinn IW, Aron JL, Pearson M, Lucas D, Reed JC (2002). The mechanism of tumor cell clearance by rituximab in vivo in patients with B-cell chronic lymphocytic leukemia: evidence of caspase activation and apoptosis induction. Blood.

[5] Krajewska M, Krajewski S, Banares S, Huang X, Turner B, Bubendorf L, Kallioniemi OP, Shabaik A, Vitiello A, Peehl D, Gao GJ, Reed JC (2003). Elevated expression of inhibitor of apoptosis proteins in prostate cancer. Clinical cancer research.

[6] Zhou S, Huang Q, Zheng S, Lin K, You J, Zhang X (2015). miR-27a regulates the sensitivity of breast cancer cells to cisplatin treatment via BAK-SMAC/DIABLO-XIAP axis. Tumour biology.

[7] Wang C, Ju H, Shen C, Tong Z (2015). miR-429 mediates delta-tocotrienol-induced apoptosis in triple-negative breast cancer cells by targeting XIAP. International journal of clinical and experimental medicine.

[8] Nestal de Moraes G, Delbue D, Silva KL, Robaina MC, Khongkow P, Gomes AR, Zona S, Crocamo S, Mencalha AL, Magalhaes LM, Lam EW, Maia RC (2015). FOXM1 targets XIAP and Survivin to modulate breast cancer survival and chemoresistance. Cellular signalling.

[9] Kluger HM, McCarthy MM, Alvero AB, Sznol M, Ariyan S, Camp RL, Rimm DL, Mor G (2007). The X-linked inhibitor of apoptosis protein (XIAP) is up-regulated in metastatic melanoma and XIAP cleavage by Phenoxodiol is associated with Carboplatin sensitization. Journal of translational medicine.

[10] Nagi C, Xiao GQ, Li G, Genden E, Burstein DE (2007). Immunohistochemical detection of X-linked inhibitor of apoptosis in head and neck squamous cell carcinoma. Annals of diagnostic pathology.

[11] Nemoto T, Kitagawa M, Hasegawa M, Ikeda S, Akashi T, Takizawa T, Hirokawa K, Koike M (2004). Expression of IAP family proteins in esophageal cancer. Experimental and molecular pathology.

[12] Fong WG, Liston P, Rajcan-Separovic E, St Jean M, Craig C, Korneluk RG (2000). Expression and genetic analysis of XIAP-associated factor 1 (XAF1) in cancer cell lines. Genomics.

[13] Yang L, Cao Z, Yan H, Wood WC (2003). Coexistence of high levels of apoptotic signaling and inhibitor of apoptosis proteins in human tumor cells: implication for cancer specific therapy. Cancer research.

[14] Cao Z, Zhang R, Li J, Huang H, Zhang D, Zhang J, Gao J, Chen J, Huang C (2013). X-linked inhibitor of apoptosis protein (XIAP) regulation of cyclin D1 protein expression and cancer cell anchorage-independent growth via its E3 ligase-mediated protein phosphatase 2A/c-Jun axis. The Journal of biological chemistry.

[15] Liu J, Zhang D, Luo W, Yu Y, Yu J, Li J, Zhang X, Zhang B, Chen J, Wu XR, Rosas-Acosta G, Huang C (2011). X-linked inhibitor of apoptosis protein (XIAP) mediates cancer cell motility via Rho GDP dissociation inhibitor (RhoGDI)-dependent regulation of the cytoskeleton. The Journal of biological chemistry.

[16] Yu J, Zhang D, Liu J, Li J, Yu Y, Wu XR, Huang C (2012). RhoGDI SUMOylation at Lys-138 increases its binding activity to Rho GTPase and its inhibiting cancer cell motility. The Journal of biological chemistry.

[17] Kleinberg L, Florenes VA, Silins I, Haug K, Trope CG, Nesland JM, Davidson B (2007). Nuclear expression of survivin is associated with improved survival in metastatic ovarian carcinoma. Cancer.

[18] Toman R (1992). Basic structural features of a lipopolysaccharide from the Coxiella burnetii strain Nine Mile in the virulent phase I. Acta virologica.

[19] Potts PR, Singh S, Knezek M, Thompson CB, Deshmukh M (2003). Critical function of endogenous XIAP in regulating caspase activation during sympathetic neuronal apoptosis. The Journal of cell biology.

[20] Galban S, Duckett CS (2010). XIAP as a ubiquitin ligase in cellular signaling. Cell death and differentiation.

[21] Huang Y, Park YC, Rich RL, Segal D, Myszka DG, Wu H (2001). Structural basis of caspase inhibition by XIAP: differential roles of the linker versus the BIR domain. Cell.

[22] Cao Z, Li X, Li J, Luo W, Huang C, Chen J (2014). X-linked inhibitor of apoptosis protein (XIAP) lacking RING domain localizes to the nuclear and promotes cancer cell anchorage-independent growth by targeting the E2F1/Cyclin E axis. Oncotarget.

[23] Crum CP, McKeon FD (2010). p63 in epithelial survival germ cell surveillance and neoplasia. Annual review of pathology.

[24] Dotsch V, Bernassola F, Coutandin D, Candi E, Melino G (2010). p63 and p73 the ancestors of p53. Cold Spring Harbor perspectives in biology.

[25] Flores ER (2007). The roles of p63 in cancer. Cell cycle.

[26] Flores ER, Sengupta S, Miller JB, Newman JJ, Bronson R, Crowley D, Yang A, McKeon F, Jacks T (2005). Tumor predisposition in mice mutant for p63 and p73: evidence for broader tumor suppressor functions for the p53 family. Cancer cell.

[27] Dohn M, Zhang S, Chen X (2001). p63 alpha and Delta Np63 alpha can induce cell cycle arrest and apoptosis and differentially regulate p53 target genes. Oncogene.

[28] Urist MJ, Di Como CJ, Lu ML, Charytonowicz E, Verbel D, Crum CP, Ince TA, McKeon FD, Cordon-Cardo C (2002). Loss of p63 expression is associated with tumor progression in bladder cancer. The American journal of pathology.

[29] Su X, Chakravarti D, Cho MS, Liu L, Gi YJ, Lin YL, Leung ML, El-Naggar A, Creighton CJ, Suraokar MB, Wistuba I, Flores ER (2010). TAp63 suppresses metastasis through coordinate regulation of Dicer and miRNAs. Nature.

[30] Melino G (2011). p63 is a suppressor of tumorigenesis and metastasis interacting with mutant p53. Cell death and differentiation.

[31] Park BJ, Lee SJ, Kim JI, Lee SJ, Lee CH, Chang SG, Park JH, Chi SG (2000). Frequent alteration of p63 expression in human primary bladder carcinomas. Cancer research.

[32] DiGiovanni J, Rho O, Xian W, Beltran L (1994). Role of the epidermal growth factor receptor and transforming growth factor alpha in mouse skin carcinogenesis. Progress in clinical and biological research.

[33] Dong Z, Birrer MJ, Watts RG, Matrisian LM, Colburn NH (1994). Blocking of tumor promoter-induced AP-1 activity inhibits induced transformation in JB6 mouse epidermal cells. Proceedings of the National Academy of Sciences of the United States of America.

[34] Huang C, Ma WY, Young MR, Colburn N, Dong Z (1998). Shortage of mitogen-activated protein kinase is responsible for resistance to AP-1 transactivation and transformation in mouse JB6 cells. Proceedings of the National Academy of Sciences of the United States of America.

[35] Huang C, Ma WY, Dong Z (1996). Requirement for phosphatidylinositol 3-kinase in epidermal growth factor-induced AP-1 transactivation and transformation in JB6 P+ cells. Molecular and cellular biology.

[36] Fang Y, Wang Y, Wang Y, Meng Y, Zhu J, Jin H, Li J, Zhang D, Yu Y, Wu XR, Huang C (2014). A new tumour suppression mechanism by p27Kip1: EGFR down-regulation mediated by JNK/c-Jun pathway inhibition. The Biochemical journal.

[37] Rossi MR, Masters JR, Park S, Todd JH, Garrett SH, Sens MA, Somji S, Nath J, Sens DA (2001). The immortalized UROtsa cell line as a potential cell culture model of human urothelium. Environmental health perspectives.

[38] Somji S, Bathula CS, Zhou XD, Sens MA, Sens DA, Garrett SH (2008). Transformation of human urothelial cells (UROtsa) by as and cd induces the expression of keratin 6a. Environmental health perspectives.

[39] Szostak E, Gebauer F (2013). Translational control by 3′-UTR-binding proteins. Briefings in functional genomics.

[40] Ha M, Kim VN (2014). Regulation of microRNA biogenesis. Nature reviews Molecular cell biology.

[41] Di Como CJ, Urist MJ, Babayan I, Drobnjak M, Hedvat CV, Teruya-Feldstein J, Pohar K, Hoos A, Cordon-Cardo C (2002). p63 expression profiles in human normal and tumor tissues. Clinical cancer research.

[42] Boominathan L (2010). The tumor suppressors p53 p63 and p73 are regulators of microRNA processing complex. PloS one.

[43] Li Y, Zhou Z, Chen C (2008). WW domain-containing E3 ubiquitin protein ligase 1 targets p63 transcription factor for ubiquitin-mediated proteasomal degradation and regulates apoptosis. Cell death and differentiation.

[44] Wang W, El-Deiry WS (2008). Restoration of p53 to limit tumor growth. Current opinion in oncology.

[45] Bosch A, Panoutsopoulou K, Corominas JM, Gimeno R, Moreno-Bueno G, Martin-Caballero J, Morales S, Lobato T, Martinez-Romero C, Farias EF, Mayol X, Cano A, Hernandez-Munoz I (2014). The Polycomb group protein RING1B is overexpressed in ductal breast carcinoma and is required to sustain FAK steady state levels in breast cancer epithelial cells. Oncotarget.

[46] Boominathan L (2010). The guardians of the genome (p53 TA-p73 and TA-p63) are regulators of tumor suppressor miRNAs network. Cancer metastasis reviews.

[47] Melar-New M, Laimins LA (2010). Human papillomaviruses modulate expression of microRNA 203 upon epithelial differentiation to control levels of p63 proteins. Journal of virology.

[48] Scheel AH, Beyer U, Agami R, Dobbelstein M (2009). Immunofluorescence-based screening identifies germ cell associated microRNA 302 as an antagonist to p63 expression. Cell cycle.

[49] Shinozuka E, Miyashita M, Mizuguchi Y, Akagi I, Kikuchi K, Makino H, Matsutani T, Hagiwara N, Nomura T, Uchida E, Takizawa T (2013). SnoN/SKIL modulates proliferation through control of hsa-miR-720 transcription in esophageal cancer cells. Biochemical and biophysical research communications.

[50] Guerit D, Philipot D, Chuchana P, Toupet K, Brondello JM, Mathieu M, Jorgensen C, Noel D (2013). Sox9-regulated miRNA-574-3p inhibits chondrogenic differentiation of mesenchymal stem cells. PloS one.

[51] Filipowicz W, Bhattacharyya SN, Sonenberg N (2008). Mechanisms of post-transcriptional regulation by microRNAs: are the answers in sight?. Nature reviews Genetics.

[52] Rodriguez A, Griffiths-Jones S, Ashurst JL, Bradley A (2004). Identification of mammalian microRNA host genes and transcription units. Genome research.

[53] Huang HY, Shariat SF, Sun TT, Lepor H, Shapiro E, Hsieh JT, Ashfaq R, Lotan Y, Wu XR (2007). Persistent uroplakin expression in advanced urothelial carcinomas: implications in urothelial tumor progression and clinical outcome. Human pathology.

[54] Fang Y, Cao Z, Hou Q, Ma C, Yao C, Li J, Wu XR, Huang C (2013). Cyclin d1 downregulation contributes to anticancer effect of isorhapontigenin on human bladder cancer cells. Mol Cancer Ther.

[55] Jia LF, Huang YP, Zheng YF, Lyu MY, Wei SB, Meng Z, Gan YH (2014). miR-29b suppresses proliferation migration and invasion of tongue squamous cell carcinoma through PTEN-AKT signaling pathway by targeting Sp1. Oral Oncol.

[56] Gao G, Chen L, Li J, Zhang D, Fang Y, Huang H, Chen X, Huang C (2014). Isorhapontigenin (ISO) inhibited cell transformation by inducing G0/G1 phase arrest via increasing MKP-1 mRNA Stability. Oncotarget.

[57] Jin H, Yu Y, Hu Y, Lu C, Li J, Gu J, Zhang L, Huang H, Zhang D, Wu XR, Gao J, Huang C (2015). Divergent behaviors and underlying mechanisms of cell migration and invasion in non-metastatic T24 and its metastatic derivative T24T bladder cancer cell lines. Oncotarget.

[58] Zhu J, Zhang J, Huang H, Li J, Yu Y, Jin H, Li Y, Deng X, Gao J, Zhao Q, Huang C (2014). Crucial role of c-Jun phosphorylation at Ser63/73 mediated by PHLPP protein degradation in the cheliensisin a inhibition of cell transformation. Cancer prevention research.

[59] Zeng X, Xu Z, Gu J, Huang H, Gao G, Zhang X, Li J, Jin H, Jiang G, Sun H, Huang C (2016). Induction of miR-137 by Isorhapontigenin (ISO) Directly Targets Sp1 Protein Translation and Mediates Its Anticancer Activity Both In Vitro and In Vivo. Mol Cancer Ther.

[60] Fang Y, Yu Y, Hou Q, Zheng X, Zhang M, Zhang D, Li J, Wu XR, Huang C (2012). The Chinese herb isolate isorhapontigenin induces apoptosis in human cancer cells by down-regulating overexpression of antiapoptotic protein XIAP. The Journal of biological chemistry.

[61] Xu Z, Zeng X, Xu J, Xu D, Li J, Jin H, Jiang G, Han X, Huang C (2016). Isorhapontigenin suppresses growth of patient-derived glioblastoma spheres through regulating miR-145/SOX2/cyclin D1 axis. Neuro Oncol.

[62] Che X, Liu J, Huang H, Mi X, Xia Q, Li J, Zhang D, Ke Q, Gao J, Huang C (2013). p27 suppresses cyclooxygenase-2 expression by inhibiting p38beta and p38delta-mediated CREB phosphorylation upon arsenite exposure. Biochimica et biophysica acta.

[63] Huang H, Ma L, Li J, Yu Y, Zhang D, Wei J, Jin H, Xu D, Gao J, Huang C (2014). NF-kappaB1 inhibits c-Myc protein degradation through suppression of FBW7 expression. Oncotarget.

[64] Cao Z, Li X, Li J, Kang B, Chen J, Luo W, Huang C (2014). SUMOylation of RhoGDIalpha is required for its repression of cyclin D1 expression and anchorage-independent growth of cancer cells. Molecular oncology.

[65] Ouyang W, Luo W, Zhang D, Jian J, Ma Q, Li J, Shi X, Chen J, Gao J, Huang C (2008). PI-3K/Akt pathway-dependent cyclin D1 expression is responsible for arsenite-induced human keratinocyte transformation. Environmental health perspectives.

